# Detection of *Cutibacterium acnes* from multiple miliary osteoma cutis

**DOI:** 10.1002/ccr3.6492

**Published:** 2022-10-20

**Authors:** Makoto Kondo, Keiichi Yamanaka

**Affiliations:** ^1^ Department of Dermatology Mie University Graduate School of Medicine Tsu, Mie Japan

**Keywords:** *Cutibacterium acnes*, multiple miliary osteoma cutis

## Abstract

The patient had a history of acne vulgaris at a young age. The excisional biopsy from the nodule of the face showed the findings of multiple miliary osteoma cutis (MMOC). As *Cutibacterium acnes* were identified in calcified nodules, *Cutibacterium acnes* may be one of the triggering factors for MMOC. MMOC patients need proper skin care because the subcutaneous calcification is slowly formed even after middle age.

## Graphical abstract

As *Cutibacterium acnes* (*C. acnes*) has been present in adult age, patients with multiple miliary osteoma cutis may reduce the risk for neoplastic growth of subcutaneous calcification, if they take care of *C. acnes* by proper caring of the facial skin.

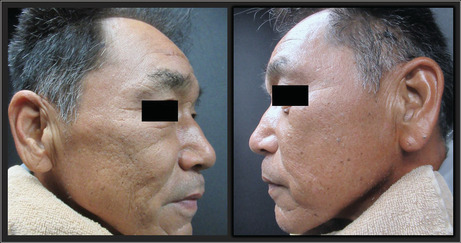

## 1 CASE

**FIGURE 1:**
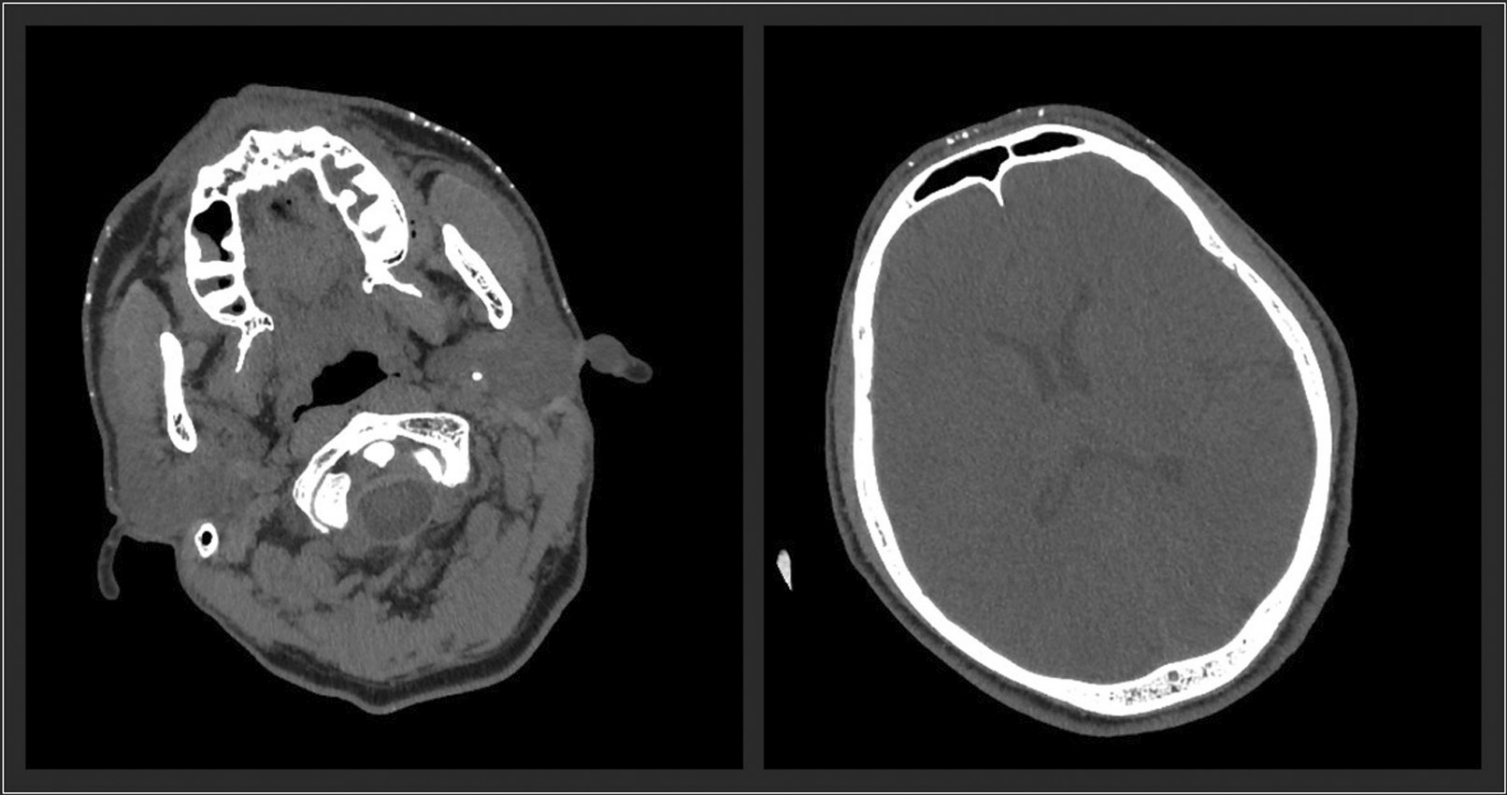
Numerous small subcutaneous calcifications are seen from the jaw to the cheeks

**FIGURE 2:**
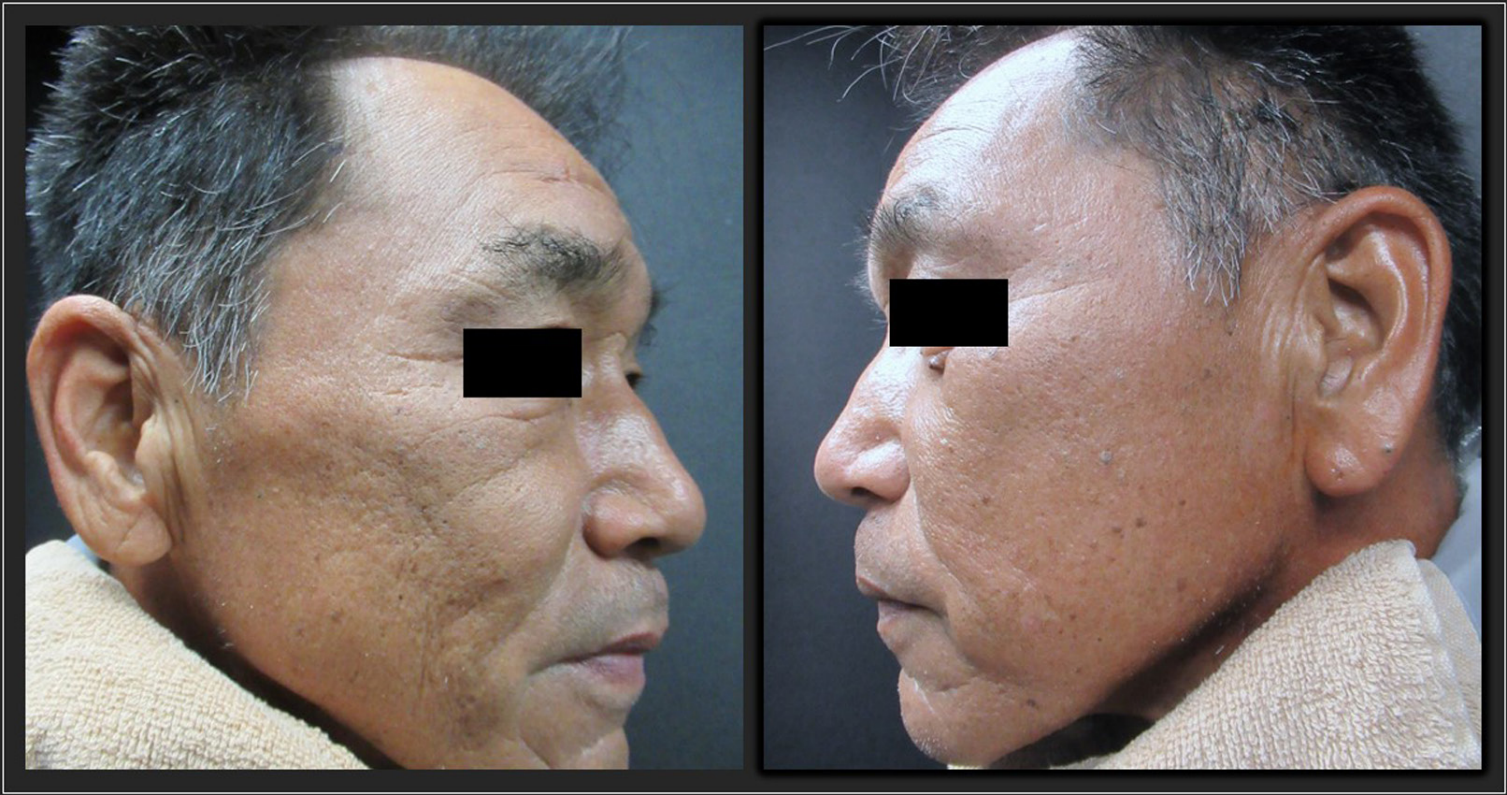
Numerous depressed scars formed on the patient's cheeks and jaw

**FIGURE 3:**
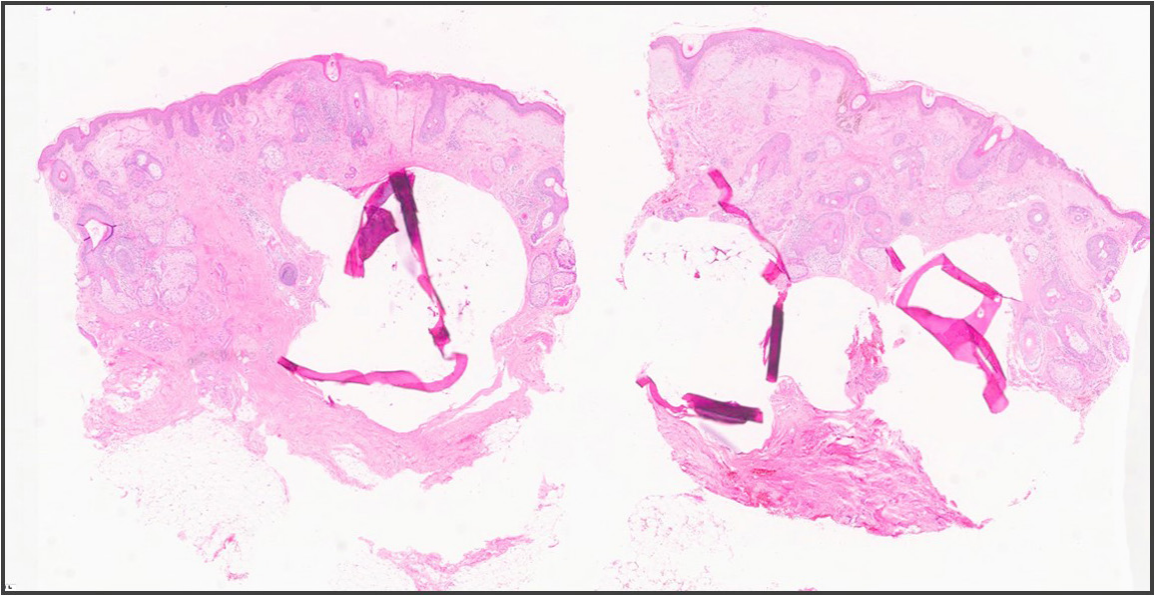
The formed osteoma in the dermis (Hematoxylin–eosin stain ×40)

A 53‐year‐old man underwent head computed tomography for a close examination of left head and neck pain. Unexpectedly, numerous calcified nodules were detected from the parietal to the mandible (Figure 1). The patient was unaware of the nodules, although the patient's face showed many depressed scars (Figure 2). The patient had a history of acne vulgaris at a young age. The excisional biopsy from the nodule showed bone structure with osteoblasts (Figure 3), and the diagnosis of multiple miliary osteoma cutis (MMOC) was made. MMOC has been reported with a history of pre‐existing acne.^1^ Therefore, by using DNA extracted from the excised tissue, the polymerase chain reaction was performed with known primers to identify *Cutibacterium acnes* (*C. acnes*).^2^ The sequence of this amplified DNA was consistent with that of *C. acnes*. The pathogenesis of MMOC is still unknown. To our knowledge, this is the first report to describe and prove the potential association between *C. acnes* and MMOC. In youth, *C. acnes* are abundant on the face, but with age, the predominant species on the face changes, and *C. acnes* declines in number. The nodules slowly increase in size after middle age.^1^ Because of the detection of *C. acnes* in subcutaneous calcified nodules, *C. acnes* may be one of the triggering factors for MMOC. MMOC patients may be able to reduce the risk for neoplastic growth of subcutaneous calcification if they take care of *C. acnes* by proper cleansing and caring of the facial skin.

## AUTHOR CONTRIBUTIONS

M.K. involved in treatment for patient and writing—original draft preparation. K.Y. involved in writing—review and editing.

## ACKNOWLEDGMENTS

The authors would like to thank the patient for giving consent for the publication of the case.

## FUNDING INFORMATION

The authors did not receive any financial support for this study.

## CONFLICT OF INTEREST

The authors declare no competing interests.

## CONSENT

Written informed consent was obtained from the patient.

## Data Availability

The datasets generated and analyzed will be available upon request to the corresponding author.
